# Modification of inhibitory control and craving through transcranial direct current stimulation as an add-on treatment for substance use disorder: protocol for a randomized controlled study

**DOI:** 10.1186/s40359-025-03506-1

**Published:** 2025-10-13

**Authors:** Sabine Vollstädt-Klein, Cagdas Türkmen, Nadja Grundinger, Alfred Wieland, Pascal-M. Aggensteiner, Ann-Kathrin Stock, Maria Stein, Franz Moggi, Florian Bublatzky, Falk Kiefer, Tobias Link, Sarah Gerhardt

**Affiliations:** 1 Department of Addictive Behaviour and Addiction Medicine, Medical Faculty Mannheim, Central Institute of Mental Health, University of Heidelberg, PO Box 12 21 20, Mannheim, 68072 Germany; 2 Mannheim Center for Translational Neuroscience, Medical Faculty Mannheim, University of Heidelberg, Mannheim, Germany; 3 German Center for Mental Health (DZPG), Partner Site Mannheim-Heidelberg-Ulm, Mannheim, Germany; 4 Department of Child and Adolescent Psychiatry and Psychotherapy, Medical Faculty Mannheim, Central Institute of Mental Health, Heidelberg University, Mannheim, Germany; 5 Cognitive Neurophysiology, Department of Child and Adolescent Psychiatry, Faculty of Medicine, TU Dresden, Dresden, Germany; 6 Translational Research Center, University Hospital of Psychiatry and Psychotherapy, University of Bern, Bern, Switzerland; 7 Department of Clinical Psychology and Psychotherapy, Institute of Psychology, University of Bern, Bern, Switzerland; 8 Department of Psychosomatic Medicine and Psychotherapy, Medical Faculty Mannheim, Central Institute of Mental Health, University of Heidelberg, Mannheim, Germany; 9 Feuerlein Centre On Translational Addiction Medicine, University of Heidelberg, Heidelberg, Germany; 10 Psychiatrisches Zentrum Nordbaden, Academic Medical Center of the University of Heidelberg, Wiesloch, Germany

**Keywords:** Substance use disorder, Alcohol use disorder, Craving, Inhibitory control, Working memory

## Abstract

**Background:**

Substance use disorders (SUDs) remain a prevalent public health issue characterized by a substantial disease burden and high relapse rates. The aim of this planned project is to investigate the optimal electrode placement and polarity of transcranial direct current stimulation (tDCS) to reduce cognitive deficits and substance craving in individuals with SUDs, thereby contributing to improved treatment outcomes, including longer abstinence periods and reduced substance use after relapse.

**Methods:**

This paper is a study protocol for a planned study. The study will enroll 162 treatment-seeking individuals aged 18 to 65 years who meet the DSM-5 criteria for alcohol use disorder (AUD), including those with other comorbid SUDs. Besides receiving treatment as usual (TAU), study participants will be randomly assigned to one of six groups: anodal stimulation over right dorsolateral prefrontal cortex (DLPFC; Group 1), left DLPFC (Group 2), or lateral occipital cortex (Group 3); sham tDCS (Group 4); computerized inhibition training (Group 5); or TAU only (Group 6). Assessments will be conducted at baseline (T1), across the directly following investigation days (T2-T4), post-intervention (T5), and at four follow-ups (after 4, 8, 12 and 24 weeks) at the Central Institute of Mental Health (Mannheim, Germany) and at the Psychiatric Center Nordbaden (Wiesloch, Germany). The primary outcomes include changes in craving and inhibitory control measured through a neuropsychological task (modified Go/No-Go task), as well as changes in electroencephalogram (EEG) activity during this task, specifically in event-related potentials including the N200 and P300 components. Secondary outcomes include abstinence days and amount of alcohol consumed.

**Discussion:**

Following the completion of this study, findings from this research could inform future therapeutic strategies for SUD, potentially advancing and complementing SUD treatment approaches by integrating tDCS as a potential relapse prevention strategy. Addressing potential challenges such as participant discomfort and high dropout rates through comprehensive support is vital for the success of this study.

**Trial registration:**

Registered at https://clinicaltrials.gov/study/NCT06959342 (Date 26.04.2025).

## Background

According to the World Health Organization, alcohol consumption leads to approximately 2.6 million deaths per year [1]. In 2019, an estimated 400 million people (7% of the population aged 15 and older) were living with an alcohol use disorder (AUD) [1, 2]. In addition, about 39.5 million people were affected by other substance use disorders (SUDs) [2], which account for approximately 9–24 of life years lost [3]. Substance use is equally widespread in the German population, thus mirroring world-wide of observations of consumption and harm. According to the 2018 Epidemiological Survey of Substance Abuse, 71.6% of respondents consumed alcohol in the past 30 days, and the 12-month prevalence of AUD was 5.9% [4]. Among other drugs, cannabis was the most common, with a 12-month prevalence of consumption of 7.1% (use disorder 1.1%), followed by amphetamines (consumption 1.2%; use disorder 0.3%) [4]. Negative consequences substance use affect not only the individual, but also our society with considerable social and economic losses. Just looking at alcohol, the US was confronted with an approximate 249 billion USD loss in 2010 [5].

Consuming a substance again after a prolonged period of abstinence is defined as a relapse in the context of a pre-existing SUD. A serious relapse is one on which the individual returns completely to previous, detrimental behavioral patterns [6]. Importantly, in individuals being treated for e.g. AUD, the approximate relapse rates range between 20–80% [7]. A relapse due to increased substance craving can be triggered by confrontation with stimuli that were previously associated with the substance [8]. Especially during protracted withdrawal, which is defined as the 4–6 weeks following last substance use, craving induced by substance-associated stimuli plays a critical role in influencing the likelihood of relapse [9]. In both regards, inhibitory control over one’s actions is vital to prevent relapse. Robust inhibitory control may help to control the compulsive drive to consume substances [10], as indicated by the sizeable percentage of patients with SUDs maintaining lifetime abstinence [11]. Recently, this observation has been translated into inhibitory control trainings as a means to improve health-related behaviors [12], such as increasing abstinent days in patients with AUD [13].

Beyond a compulsion to consume a substance, SUD chronically progresses. Affected individuals lose control over regulating substance consumption [14, 15]. Later, consumption is motivated mainly by a desire to evade withdrawal symptoms if use were to be ceased [16]. At the neuronal level, the prefrontal cortex mediates healthy executive functioning that shifts to striatal control over drug-taking behavior in the course of voluntary to compulsive drug use [17]. Due to a chronic substance consumption, SUDs lead to neurobiological impairments in regions relevant for executive functions, episodic memory, visuospatial capacities [18], and inhibitory control over the desire to consume [19, 20]. Also, structural abnormalities in the prefrontal cortex of substance-dependent individuals were observed, like reduced grey matter volume and density in the prefrontal cortex [21–23]. These micro- and macro-structural abnormalities translate into an altered recruitment of prefrontal brain regions and less connectivity with the striatum, which leads to impairments in inhibitory control [24–26]. In participants wanting to quit smoking, Berkman et al. [27] observed less activation in the right frontal cortex during a response inhibition task which was associated with more cigarettes smoked. Higher activation, on the other hand, was related to a reduced coupling between craving and cigarettes smoked. The authors concluded that a higher baseline-response inhibition, as seen in the activation of the right frontal cortex, might have a real-life effect on addictive behavior. In AUD, heightened activation in prefrontal regions during a working memory task might be relevant for executive control and can be seen as a resilience factor regarding possible relapse [28].

Within the prefrontal brain regions, the dorsolateral prefrontal cortex (DLPFC) plays a vital role in top-down regulation of substance craving and substance-taking behavior (inhibitory control) [29]. Impaired control over adequate action selection was associated with reduced activity in the DLPFC [30]. Functional imaging studies also suggest a shift of activation from the ventral to the dorsal striatum associated with lower cortical control when viewing alcohol cues [31], which can be reversed through cue-exposure based extinction training [32]. Ongoing studies use neurofeedback to target these abnormal activation patterns (e.g., striatal hyperactivation or prefrontal hypoactivation) [33]. Other approaches to strengthen cortical inhibitory control over limbic reward-driven behavior include approach-avoidance and inhibition trainings [34–36]. In addition, event-related brain potentials (ERP) derived from electroencephalography (EEG) provide valuable biomarkers in the study of executive functions or incentive salience in SUDs [37]. For example, alterations in ERPs such as the fronto-central N200 (a negative going waveform at 200–300 ms) and the central-parietal P300 (a positive going waveform at 300–500 ms) components have been associated with alcohol use and inhibitory control [38] and predicted relapse [39, 40]. In SUD, during different neuropsychological tasks assessing inhibitory control and error processing, this was observed as less pronounced amplitudes for the N200 and P300 components [41].

In sum, SUDs play a major role within the field of mental disorders and is are marked by a chronic course, including loss of inhibitory control over the consumption, increased substance craving, and repeated relapses. Whereas the prefrontal cortex mediates healthy, executive functioning during the controlled consumption of drugs, a shift to a striatal control represents the compulsive drug-taking habit [17]. According to this view, a loss of cognitive control (or in other words, a weakened inhibitory control) hinders behavioral control of impulses triggered by substance craving due to changes in brain structure and functioning are fundamental to the development of addiction.

### Transcranial direct current stimulation as a potential neuroenhancer in SUD

Transcranial direct current stimulation (tDCS) involves the induction of a constant low-amperage electric current applied to the cortex via surface electrodes positioned on the scalp of the subject. It can be used to enhance or suppress cortical plasticity in the human cortex [42]. The electric current flows from the positively charged anode towards the negatively charged cathode. Consequently, cortical excitability is expected to be enhanced under the anode, and decreased under the cathode [43]. The effect of tDCS on a specific region is determined by the polarity of the stimulation, the frequency of stimulation sessions, and density and duration of the current penetrating the skull [44]. Initially applied to the motor cortex [43, 45], tDCS was later used to target other brain regions in healthy individuals to enhance cognitive functions such as executive performance [46] and working memory [47]. Meta-analyses report that higher intensities and/or longer stimulation durations may amplify these effects in healthy individuals as well as those with mental disorders [48–51].

In the purpose of the present study, we assess the neuroenhancing effects of tDCS to improve the treatment of SUD. Specifically, we aim to apply tDCS in order to increase inhibitory control and thus reduce substance craving in SUD patients. When studying SUD, the DLPFC should be addressed, as this region is involved in (a) decision making, inhibitory control, attentional bias and awareness, and (b) cue-induced or spontaneous craving, and further (c) affects the reward circuitry via efferent connections to the nucleus accumbens and ventral tegmental area [17, 27, 52].

Regarding inhibitory control, tDCS can improve Stroop task reaction times when stimulating the left DLPFC in healthy young adults [53], but findings are mixed in populations with impaired inhibitory control such as attention deficit/hyperactivity disorder. Some studies found an improved inhibitory control after a single session of tDCS over the left DLPFC [54, 55], while others did not [56]. No standardized protocol exists for enhancing inhibitory control via DLPFC stimulation and outcomes appear to be influenced by current direction, including in SUD populations. In individuals with cocaine use disorder, both left and right anodal DLPFC stimulation resulted in a reduction of risky behavior [57] in the balloon analog risk task. However, in the game of dice task, the subjects’ performance depended on laterization: right anodal stimulation reduced risky behavior, while left anodal stimulation increased risky behavior [57]. A similar lateralization-dependent pattern has been observed in chronic marijuana users in a decision-making paradigm [15].

Results of functional imaging studies in SUD are in line with previous studies using multiple tDCS sessions with anodal stimulation over the right DLPFC. Placing the anode over the right DLPFC could elevate activation in this area and lead not only to an improved response inhibition in experimental paradigms, but may also improve real-world forms of response inhibition. However, results on tDCS as an add-on treatment in SUD are still mixed or inconclusive [52]. Regarding substance craving, studies on both left and right anodal DLPFC stimulation have yielded mostly promising results so far for alcohol [58–61], methamphetamine [62], and nicotine [63–66]. Most of these studies only used a single tDCS session, but multiple stimulation sessions (e.g. at least five) seem to result in more stable and longer lasting effects, as measured by time to first relapse [44, 59, 61, 63, 67–69]. In contrast to a sham stimulation, anodal stimulation of the right DLPFC on five consecutive days leads to a rapid reduction of craving in individuals with SUDs when comparing craving of the first to the last stimulation day [68]. Klaus et al. [61] were able to report a median survival time (i.e. time to first relapse) four times longer in the active tDCS group receiving anodal stimulation over the right DLPFC compared to sham stimulation. Interestingly, in alcohol and crack use disorder, effects of multiple tDCS sessions with the electrodes placed over the DLPFC (anodal right) were also observed regarding changes in the event-related potential of the P300 amplitudes in response to substance cues [70]. Further, a positive effect of tDCS on electrocortical (P300 but not N200 components) and behavioral (inhibitory control) measures might be long-lasting [71].

Summarizing previous research, tDCS is recommended as a potential treatment for enhancing specific cognitive performances. Results from work on tDCS in SUD imply that improvement of inhibitory control is more likely after anodal stimulation over the right DLPFC. There are promising results for reduced craving after stimulation over both hemispheres, with more long-term effects following anodal stimulation over the right DLPFC, as well. Lastly, multiple stimulation sessions seem to be more effective than a single session.

Building upon these sparse but promising results of tDCS in SUD as an add-on treatment [52], further research should clarify the questions of (1) laterality of DPFC tDCS stimulation, (2) the influence of tDCS on brain metabolism in general, and a comparison to (3) a computerized inhibition training and (4) sham tDCS as well as treatment as usual, on substance craving, inhibitory control, and abstinence.

### Objectives

Previous research has identified impaired inhibitory control and increased substance craving as key features of SUD, with evidence suggesting that tDCS over the DLPFC can improve executive functions and reduce craving in individuals with SUDs [72, 73]. However, the effects of tDCS seem to be hemisphere-dependent and placebo effects may confound these findings [74].

The proposed study aims to investigate inhibitory control in patients with SUD using computer-based neuropsychological assessments before and after administering five tDCS sessions as compared to an inhibition training or TAU alone. TAU will be a qualified detoxification program. Additionally, the effect of tDCS on inhibitory control will be assessed using EEG recordings, with a focus on the N200 and P300 components. Participants with a main diagnosis of AUD, including those with additional SUDs, will be included. We did not limit the sample to one specific SUD, as excluding certain substances would reduce the generalizability of the results and transferability in the clinical routine. Six groups will be compared: three tDCS conditions, one computerized inhibition training, one sham tDCS group, and one group with treatment as usual only. Multiple sham tDCS could already have a direct, neurobiological effect [75]. Hence, we decided to include two active control conditions (stimulation over the visual cortex and an inhibition training) and one sham tDCS condition.

First, anodal stimulation over the right and the left DLPFC will be compared. For this purpose, stimulation will take place over the F3 (left DLPFC) and F4 (right DLPFC) electrode positions according to the EEG 10–20 system to determine whether inhibitory control is indeed hemisphere-sensitive while craving reduction is not. This would constitute an influencing factor that future studies should consider. If stimulating or inhibiting the DLPFC in the ‘wrong’ direction (i.e., anodal left) indeed increases risky behavior, this might lead to distorted results when the outcome measure is, for example, time to first relapse, even though craving might have been reduced by the stimulation (left and right DLPFC: group 1 and group 2).

Second, due to the poor spatial resolution of tDCS (with one anodal and one cathodal electrode), we will include a control group with the anode over O1 (occipital cortex borderline to the cerebellum) and cathode over Cz to account for potential global effects on brain metabolism and task performance (occipital: group 3).

Third, we further include a computerized inhibition training to examine effects of an active, highly frequent contact and behavioral training compared to tDCS (inhibition training; group 5).

Forth, to avoid confounds related to placebo or practice effects in the neuropsychological assessment, participants following the same protocol with either sham tDCS or without receiving any intervention will be included to control for placebo effects of tDCS and the study participation being a ‘treatment’ by itself (sham: group 4, treatment as usual, TAU: group 6).

As we are interested in whether the tDCS add-on has a superior positive impact on treatment outcome, we will follow up participants for 24 weeks to record relapse timing and frequency.

In summary, tDCS is a promising tool as a neuroenhancer and add-on therapy in SUD. It is non-invasive, safe to use, and can be easily and widely applied and does not require well-equipped clinical or research facilities to be of beneficial use. If successfully reducing substance craving and increasing abstinence rates in our study sample, tDCS may be considered universal to all types of SUD, thus greatly advancing treatment options for individuals with SUDs. We aim to investigate the most effective way to simultaneously improve inhibitory control and craving using tDCS. When proven superior to the control conditions (sham tDCS, tDCS over visual cortex and inhibition training), our results may contribute to developing a gold standard for its clinical application.\

### Hypotheses

#### Primary: effects of tDCS on inhibitory control and substance craving

1. Anodal tDCS over the right DLPFC as an add-on-therapy improves inhibitory control in SUD in comparison to anodal stimulation over the left DLPFC or occipital cortex, inhibition training, sham stimulation or TAU alone at T5 and T8.
2. Anodal tDCS over the right DLPFC as an add-on-therapy increases fronto-central N200 and central-parietal P300 ERP amplitudes for substances cues and inhibitory control during task-associated EEG measurements at T5 and T8.
3. Anodal tDCS over the right as well as the left DLPFC as an add-on-therapy reduces craving in SUD in comparison to stimulation over the occipital cortex, inhibition training, sham stimulation or TAU alone from T1 to T9.

#### Secondary: effects of tDCS as an add-on treatment in SUD

4. Anodal tDCS over the right DLPFC as an add-on treatment positively influences the treatment outcome (defined as abstinence or harm reduction) for SUD patients in comparison to stimulation over the occipital cortex, inhibition training, sham stimulation or TAU alone. Therefore, during the follow-up period (T6-T9), time to first relapse (treatment outcome: duration of abstinence) and amount of substance consumed (treatment outcome: harm reduction) is expected to be longer and less, respectively.

## Methods

### Sample size

The sample size estimation in based on the above-listed primary directed hypotheses. According to previous results on craving and inhibitory control [53, 54, 58, 60, 62], the effect size is estimated to be small to medium, f = 0.15. A sample size calculation was performed using the software G*Power http://www.gpower.hhu.de), which yielded 25 participants per group (power 80%, alpha 5%. repeated measures ANOVA and within-between interaction). In order to achieve this sample size, a total of 162 individuals (27 per group) will be assessed, whereby data losses are assumed (e.g., due to a lack of compliance, hardware failure).

### Inclusion and exclusion criteria

#### Inclusion criteria

- main diagnosis: alcohol use disorder according to DSM-5
- patients of any gender aged 18 to 65
- normal vision or correctable visual impairment
- sufficient ability to communicate verbally and in writing
- ability to give fully informed consent after reviewing thorough written information

#### Exclusion criteria

- withdrawal of consent
- severe internal, neurological, or psychiatric comorbidities (e.g., lifetime schizophrenia, bipolar disorder, or other severe mental disorders according to ICD-10 and DSM-5, such as severe depression or PTSD within the last 12 months)
- Exclusion criteria for an EEG/tDCS examination (e.g. metal implants in the head, epilepsy, etc.)
- severe withdrawal symptoms (Revised Clinical Institute Withdrawal Assessment for Alcohol Scale, CIWA-R > 7) [76] at baseline assessment
- alcohol intoxication (breath alcohol concentration > 0 ‰) at any examination time point
- Pharmacotherapy with psychoactive substances within the last 14 days (exceptions: clomethiazole or benzodiazepines used in withdrawal treatment, provided they were discontinued at least 3 days prior; antidepressants or anxiolytics taken at stable doses)
- drug or alcohol use within the last 7 days during add-on treatment period (T1-T5)
- for women: pregnancy
- suicidal tendencies or danger to others

### Experimental procedure

*N* = 162 SUD patients, who voluntarily enter a qualified detoxification treatment program, will be examined (inpatient or day-clinic setting) at the Department of Addictive Behaviour and Addiction Medicine at the Central Institute of Mental Health in Mannheim, Germany and the Clinic for Addiction Therapy and Withdrawal at the Psychiatrisches Zentrum Nordbaden in Wiesloch, Germany. A randomized assignment (block randomization with permuted blocks of size 12) to one of six groups will be performed. All participants will be included during their second week of hospital stay to ensure completed detoxification (at least 7 days of abstinence) and enrollment in the treatment as usual (TAU). TAU comprises an at least three weeks long therapy duration including medical and therapeutic treatment. We will follow a placebo-controlled single-blind study design, in which participants do not receive any information about electrode placement or active vs. sham condition, except in the non-tDCS groups, namely group 5 (inhibition training) and group 6 (TAU).

**Fig. 1:**
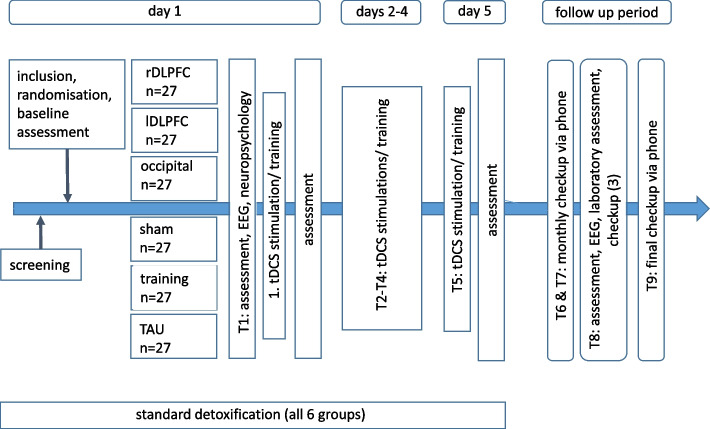
Study design including four follow-ups over a period of 6 months

Four groups will receive 20-min tDCS sessions using the Sooma Duo™ device (Sooma Oy, Helsinki, Finland) over five consecutive days at an intensity of 2 mA (≈0.08 mA/cm^2^). Electrodes (25 cm^2^) will be placed using a standardized EEG cap following the 10–20 system to ensure consistent positioning across sessions [44]. The current will be ramped up from 0.3 mA to 2 mA at 0.1 mA/s, maintained for 20 min during active stimulation. Stimulation sites will differ by group:

- Group 1: Anode over right DLPFC (electrode position F4), cathode over left DLPFC (electrode position F3)
- Group 2: Anode over left DLPFC (electrode position F3), cathode over right DLPFC (electrode position F4)
- Group 3: Anode over occipital cortex (electrode position O1), cathode over electrode position Cz.
- Group 4 will receive sham stimulation (anode over electrode position F3, cathode over electrode position F4). For blinding, the current will ramp up to 2 mA and then immediately ramp down to 0 mA, mimicking the sensation of active stimulation while avoiding physiological effects. With respect to sham tDCS as a control condition, a strong and possibly heterogonous placebo effect is still under discussion. This effect may also be due to using short, active stimulations at the beginning of a sham tDCS that could induce neurophysiological changes [75]. However, an active sham condition will improve blinding of the participants towards the intervention as switching off the current does not represent a reliable sham [77].
- Group 5 receives a computerized inhibition training using a go-/no-go-task with individually adapted, alcohol-related stimuli, as described in Stein et al. [13].
- Group 6 receives only TAU but follows the same study protocol with respect to all measurement timepoints; however, there is no contact from T2 and T4. During the follow-up period of 24 weeks, a total of one personal and three telephone contacts are planned. All participants will be contacted after 4, 8, and 24 weeks via telephone and possible relapses and amount of substances consumed will be assessed in a self-reported manner. For the third follow-up after 12 weeks, all participants will be invited for a personal follow-up assessment (timepoint T8). See Figure 1 for further details.

On the *first day* (T1/baseline assessment; e.g. Monday), all individuals are comprehensively informed about the goals of the proposed study and all study-related measures. Individuals will give their written informed consent and will receive a copy of the relevant study information and consent agreement. Subsequently, diagnostic interviews will be performed to assess eligibility for study participation, pre-existing conditions (mental, neurological and somatic in nature), validate the diagnoses of SUD, and assess current consumption habits. Pregnancy and drug tests will also be completed. Eligible participants will be asked to complete questionnaires and computer-based neuropsychological assessments (see Table 1). Participants will receive an EEG recording during one part of the neuropsychological assessment, namely during a modified Go/No-Go paradigm including various working memory loads [78] to assess the interaction between working memory load and response inhibition programmed in Presentation® software (Neurobehavioral Systems, Inc., Berkeley, CA, USA). At the Wiesloch study site, EEG recordings are conducted using a mobile EEG system (24 channels with active electrodes; sampling rate 1000 Hz; mBrainTrain, Belgrade, Serbia). At the Mannheim site, stationary EEG assessments are performed using BrainVision Recorder software package (32 channels with active electrodes; sampling rate 1000 Hz; Brain Products GmbH, Gilching, Germany). Two channels are occupied by the tDCS electrodes for both systems (for groups 1,2 and 4: F3 and F4; for group 3: Cz and O1). After completing all assessments, participants receive their first add-on treatment. For the first tDCS session, the tDCS study groups (groups 1 to 3) will receive a 20-min long tDCS with 0.08 mA/cm2, over the respective brain areas. The inhibition training group will perform a go-/no-go training according to Stein et al. [13] and the sham group will receive a 20 min long sham tDCS. Before and after the stimulation or training, craving will be assessed (see Table 1).

**Table 1:** Measurements and interventions

		T1	T2-T4	T5	T6-7	T8	T9
Baseline information and medical screening	Sociodemographic data	X					
	Internistic, neurological examination	X					
	Drug/pregnancy urine screening	X		X		X	
	Menstrual cycle	X					
	Structured Clinical Interview for DSM-5	X					
	Edinburgh Handedness Inventory (EHI, [79])	X					
Questionnaires Alcohol/Substance	Craving Scale [80] Alcohol Urge Questionnaire (AUQ, [81])	X X	X X	X X	X X	X X	X X
	Fagerstrøm Test of Cigarette Dependence (FTND, [82])	X				X	
	Craving Automated Scale for Alcohol (CAS-A, [83])	X				X	X
	Obsessive Compulsive Drinking Scale (OCDS, [84])	X					
	Alcohol Dependence Scale (ADS, [85])	X					
Questionnaires Clinic	Beck Depression Inventory–II (BDI-II [86]) Quality of life [87]	X X		X		X X	
	Perceived Stress Scale (PSS, [88])	X				X	
	Barratt Impulsiveness Scale (BIS, [89])	X		X		X	
	Creature of Habit Scale (COHS, [90])	X				X	
	ASRS-V1.1 Screener (WHO, 2004) Childhood Trauma Questionnaire (CTQ, [91])	X X					
Outcome consumption	Form 90 interview [92]	X		X	X	X	X
	Time to relapse	X		X	X	X	X
	Percentage of abstinence/heavy drinking days	X		X	X	X	X
Neuropsychological assessment	Delay Reward Discounting Task [93]	X		X		X	
	Dimensional Card Sorting Task [94]	X		X		X	
	Dot-probe task [95]	X		X		X	
	Stop-signal task [96]	X		X		X	
Electroencephalography (EEG)	Modified Go/NoGo paradigm [78]	X		X		X	
Interventions	TAU + active tDCS (Groups 1–3)	X	X	X			
	TAU + sham tDCS (Group 4)	X	X	X			
	TAU + computerized inhibition training (Group 5) TAU (Group 6)	X X	X X	X X			
	Adverse events	X	X	X			

*T1* = *first investigation day, T2-4* = *second to fourth investigation days, T5* = *fifth investigation day, T6-7* = *follow up after 4 weeks (T6) and 8 weeks (T7) *via* phone, T8* = *follow up after 12 weeks on site, T9* = *follow up after 24 weeks *via* phone*

#### Days 2 to 4 (T2-T4; duration ca. 40 min)

Participants will either continue with TAU (group 6), receive a 20-min long tDCS (groups 1–3), a ca. 20-min inhibition training (group 4), or a 20 min long sham tDCS (group 5) for each of the following three days (e.g., Tuesday, Wednesday, Thursday) in addition to their standard treatment. Craving will be assessed after each session. Adverse events are systematically assessed throughout the add-on treatment period. At the beginning of each investigation day, participants are asked about any adverse events that occurred since the previous session.

#### Day 5 (T5; duration ca. 1 h)

Participants will receive the fifth and last 20-min long tDCS (groups 1 to 3), sham tDCS (group 4), or an inhibition training (group 5) (e.g., Friday). Following, the same computer-based neuropsychological assessment will be completed (T3), and craving will be re-assessed. A full list of assessments is provided in Table 1. Adverse events that occurred since T4 are documented at the beginning of the investigation day. As participants are not contacted again until 28 days after T5 for the first follow-up assessment, any adverse events related to the final session are assessed and documented at the end of the T5 assessment.

#### Follow-up 1 (T6), 2 (T7) and 4 (T9; duration ca. 10 min)

All participants (groups 1 to 6) will be asked to answer questions regarding (possible) relapse and amount of substance consumed using REDCap electronic data capture tools hosted at the Central Institute of Mental Health [97, 98]. Subsequently, they will complete an interview via telephone four, eight and 24 weeks after T5. Relapse and amount of substances consumed will be recorded. Craving will also be assessed. A full list of assessments is provided in Table 1.

#### Follow-up 3 (duration ca. 60 min; time point T8)

For the third follow-up interview after 12 weeks, all participants will be invited to come to the institute again to complete the above mentioned, computer-based neuropsychological assessment (T8). A second EEG recording will take place during the same neuropsychological task. Further, relapse and amount of substances consumed as well as craving will be assessed again. A full list of assessments is provided in Table 1.

### Statistical analyses

The behavioral and EEG results of the inhibitory control paradigm [78] and craving ratings are defined as primary outcome measures. The results of the inhibitory control paradigm will be compared between testing days before (T1) and after fifth (T5) stimulation or training and after a follow-up period of 12 weeks (T8). Craving rates will be compared before and after the first stimulation/training (T1) and across the subsequent stimulation/training sessions (T2-T5), as well as during the follow-up period (T6-T9). Secondary outcomes include duration of abstinence and amount of substance consumed during the follow-up period.

Regarding the EEG data, resampling (to 250 Hz), filtering (70 Hz high- and 0.1 Hz low-pass filter; 50 Hz notch filter), re-referencing to an average reference, manual inspection, independent component analyses, and segmentation (from –200 to 1200 ms) will be conducted. Removed channels due to technical and regular artifacts (e.g., movement artifacts, excessive noise) will be interpolated using neighboring electrodes. ERP components will be scored as mean amplitudes between 200–300 ms and 300–500 ms over fronto-central and central-parietal electrodes for the N200 and P300 components, respectively. All data (EEG, neuropsychological and psychometric data) will be analyzed using t-tests, repeated measures analysis of variance (rmANOVA) and Pearson correlations. The influence of treatment modality (tDCS add-on intervention over various locations versus inhibition training versus standard treatment) on relapse will be tested with a Cox Regression (outcome variable: time until first relapse). Due to the included individuals and the matched-group design, potential influencing factors on primary and secondary endpoints (e.g., type of substance, severity of SUD, number of comorbidities) will be included in the analyses as covariates. In secondary analyses, we will also take a closer look at the potential influence of these factors on treatment response. In this way, it might be possible to identify subgroups that respond particularly well to the treatment (‘treatment responders’) with the goal of precision-targeted treatments for addiction. Statistical analysis will be conducted using SPSS (IBM Corp., Armonk, N.Y., USA).

### Risks and strengths of the study

Risks regarding successful study completion primarily include dropouts and relapse. Study completion will be possible in case of relapse, to not only reduce the threshold for participants to keep in touch, but to additionally examine the aspect of harm reduction, namely reduced substance consumption in the absence of abstinence. Stepwise reimbursement is intended to support participation during the follow-up period.

The advantages of this study outweigh the risks. tDCS is a mobile, low-cost, and easy-to-administer technique, with potential for at-home use post-discharge. Devices enabling self-stimulation of the DLPFC are already available and require no specialized training. Notably, tDCS has been established as a safe, well-tolerated adjunct treatment for depression [99], and may hold promise for SUD treatment. However, more evidence is needed to support its clinical application in an addiction context. Although alcohol-withdrawing individuals may have a lower seizure threshold [100], a review of tDCS safety has not identified any serious adverse effects for conventional tDCS protocols (≤ 40 min, ≤ 4 mA, ≤ 7.2 Coulombs), even in neurologically vulnerable populations such as those with stroke or epilepsy [101].

Examining individuals with different kind of SUDs allows drawing conclusions regarding general (neuro-)biological mechanisms and treatment options for a large body of affected individuals, not only a small subgroup with a specific SUD. In addition, we will be able to investigate in a large heterogeneous sample whether there are individual factors that influence the treatment response in order to analyze whether the one-size-fits-all approach is appropriate here or whether the approach is particularly suitable for a subset of patients.

### Ethical and legal aspects to this study

This experimental study comprises standard treatments and diagnostic routines. tDCS is generally very well tolerated with little to no side effects. A stimulation with tDCS can be perceived as unpleasant. The manufacturer (https://soomamedical.com/en/science-tdcs/#safety) reported itching, short-term headaches and skin irritations. Medical doctors will be present on site and available to respond to any issues that may occur during tDCS application. EEG examinations are overall a safe procedure; however, temporary skin irritation may occur due to the application of electrode gel. Inclusion and exclusion criteria and procedures, as well as a professional examination and maintenance of the instruments will help to prevent these possible side effects. All procedures comply with the declaration of Helsinki. Written informed consent will be obtained. The study as presented here has been ethically approved by the ethics committee of the Medical Faculty Mannheim, Heidelberg University, Germany (2018-561N-MA) and by the State Medical Council of Baden-Württemberg (B-F-2024–088) as well as subsequent amendments. Data collected during this period will be managed at the Central Institute of Mental Health in Mannheim, Germany, and will be exclusively used for research purposes outlined in the proposed study. Participant information, data recording and storage will be handled in accordance with the General Data Protection Regulation as passed by the European Union.

## Discussion

Recent meta-analytic evidence shows that tDCS holds promise as a low-risk intervention for reducing substance craving, with right anodal DLPFC placement exerting more pronounced effects on craving reduction [73]. However, the current body of evidence is limited by the lack of direct comparisons between different anodal electrode placements as well as scarce research examining cognitive outcomes and underlying neurobiological mechanisms, which the present study aims to address. Given the central role of craving in relapse [102], integrating tDCS into existing SUD intervention and prevention strategies could provide a valuable tool for relapse prevention.

To ensure the success of the study, it will be critical to address common practical challenges. Participant discomfort, such as fatigue resulting from five consecutive investigation days, as well as high dropout rates frequently encountered in SUD treatment studies [103], will be proactively mitigated through regular check-ins and individualized support throughout the intervention, as needed. Qualified physicians and psychotherapists will be available to respond to any issues that arise to help create a supportive environment.

## Data Availability

No datasets were generated or analysed during the current study.

## Abbreviations

ANOVA: Analysis of variance
AUD: Alcohol use disorder
DLPFC: Dorsolateral prefrontal cortex
DSM: Diagnostic and Statistical Manual of Mental Disorders
EEG: Electroencephalography
ERP: Event-related potential
ICD: International Classification of Diseases
PFC: Prefrontal cortex
SUD: Substance use disorder
TAU: Treatment as usual
tDCS: Transcranial direct current stimulation
